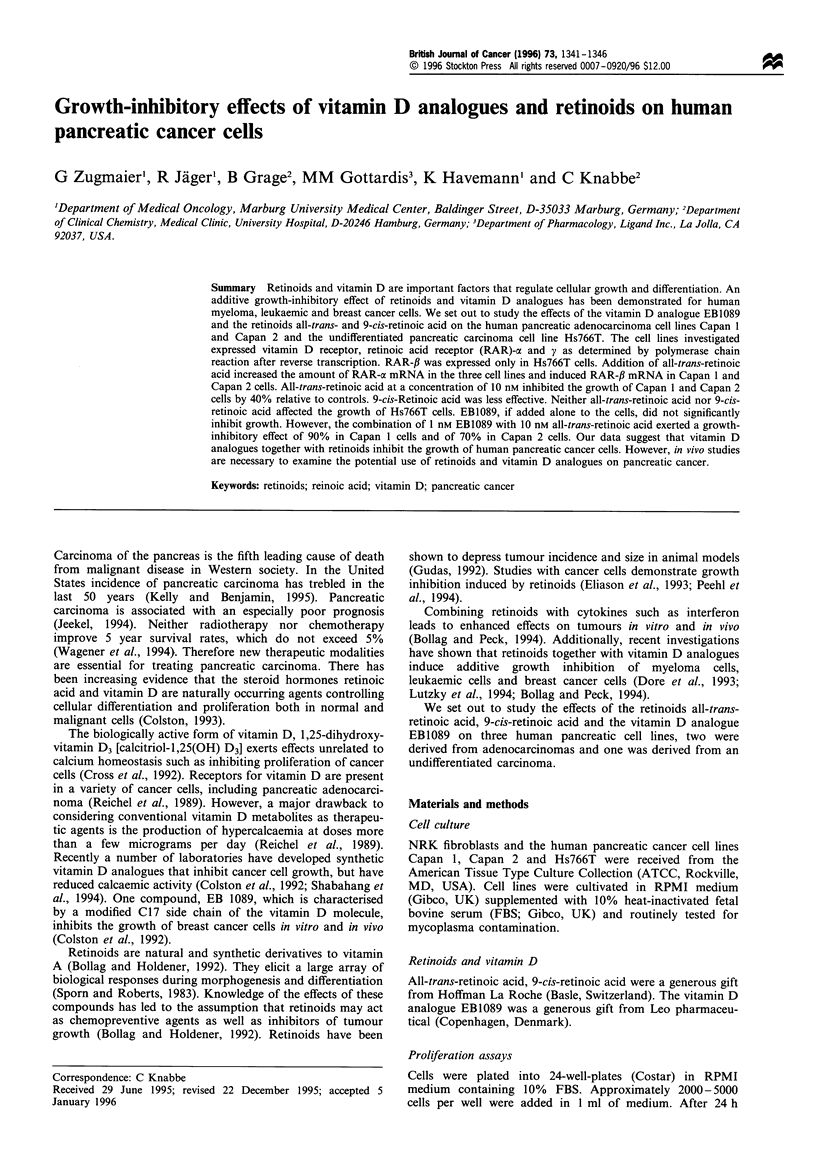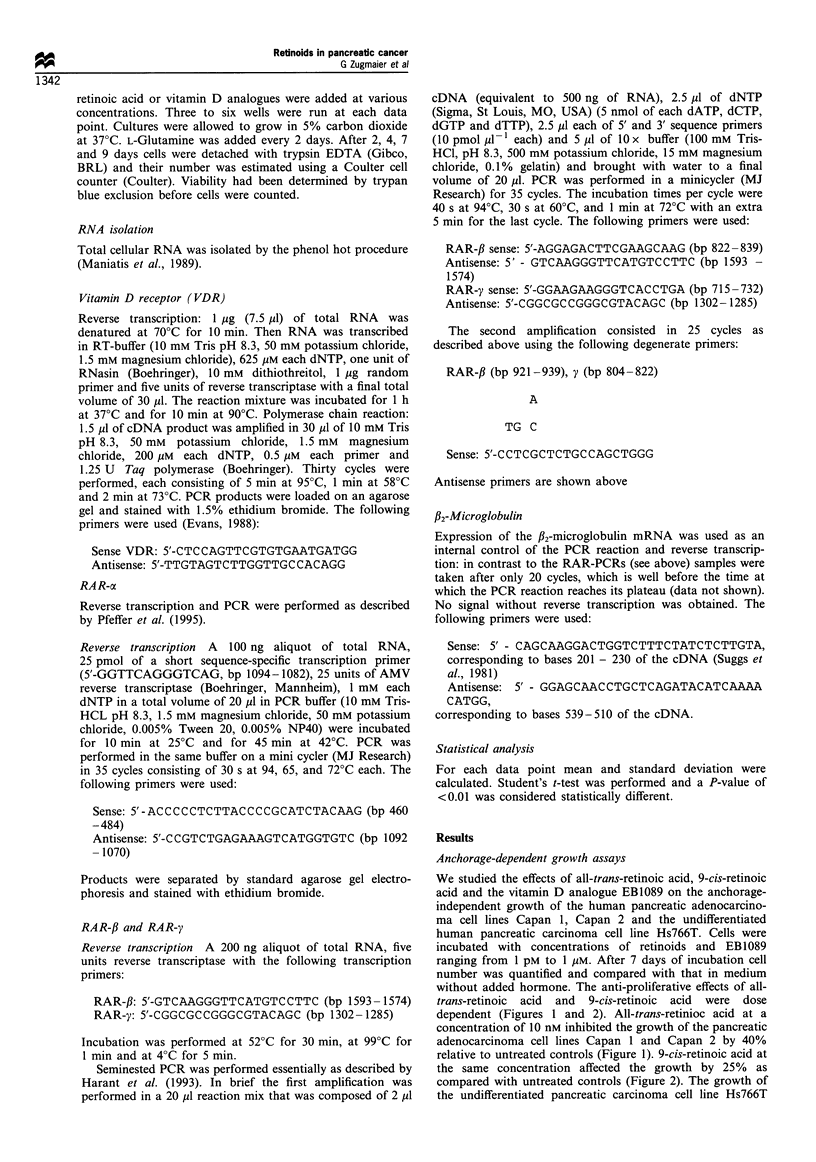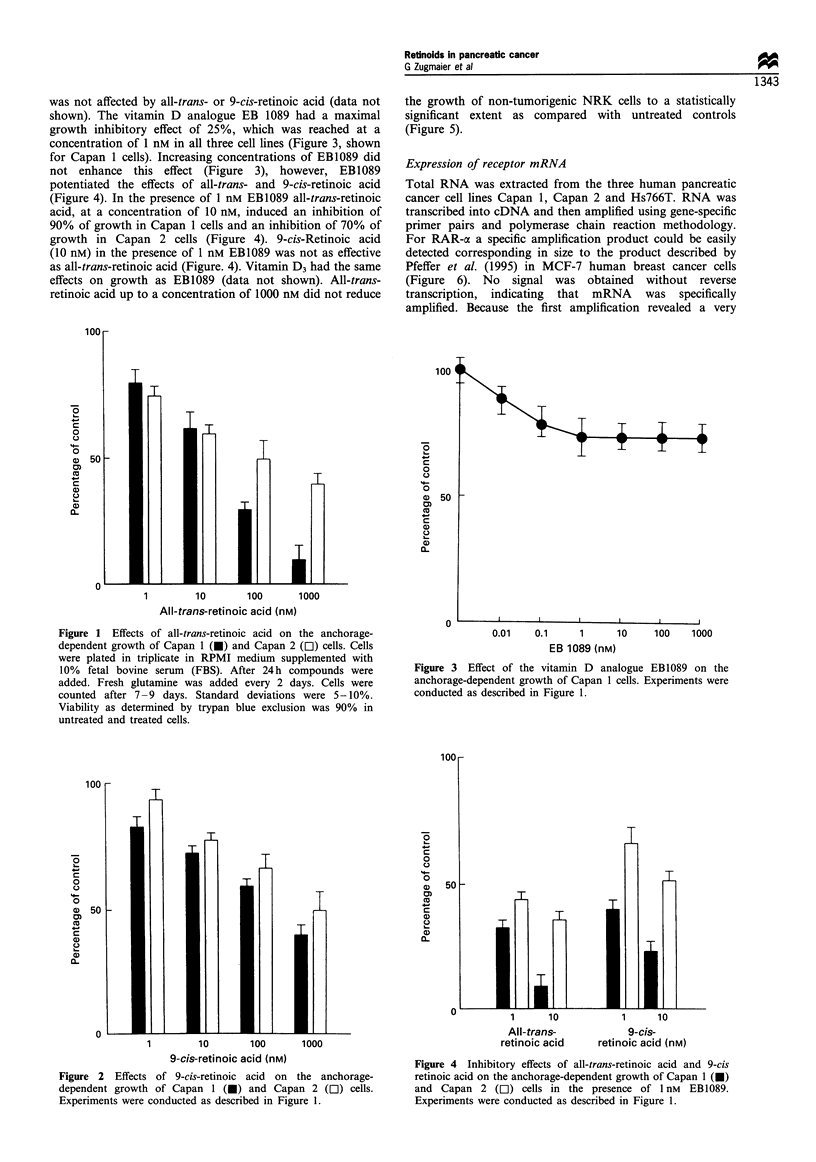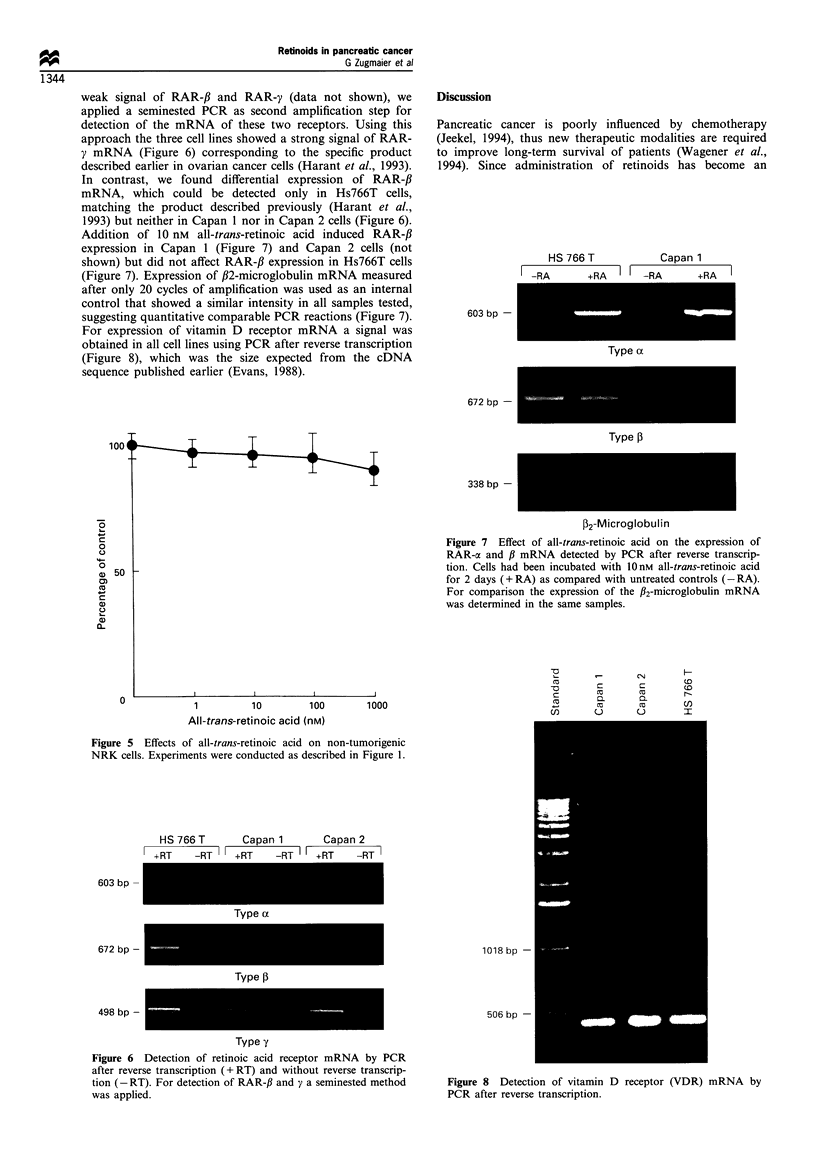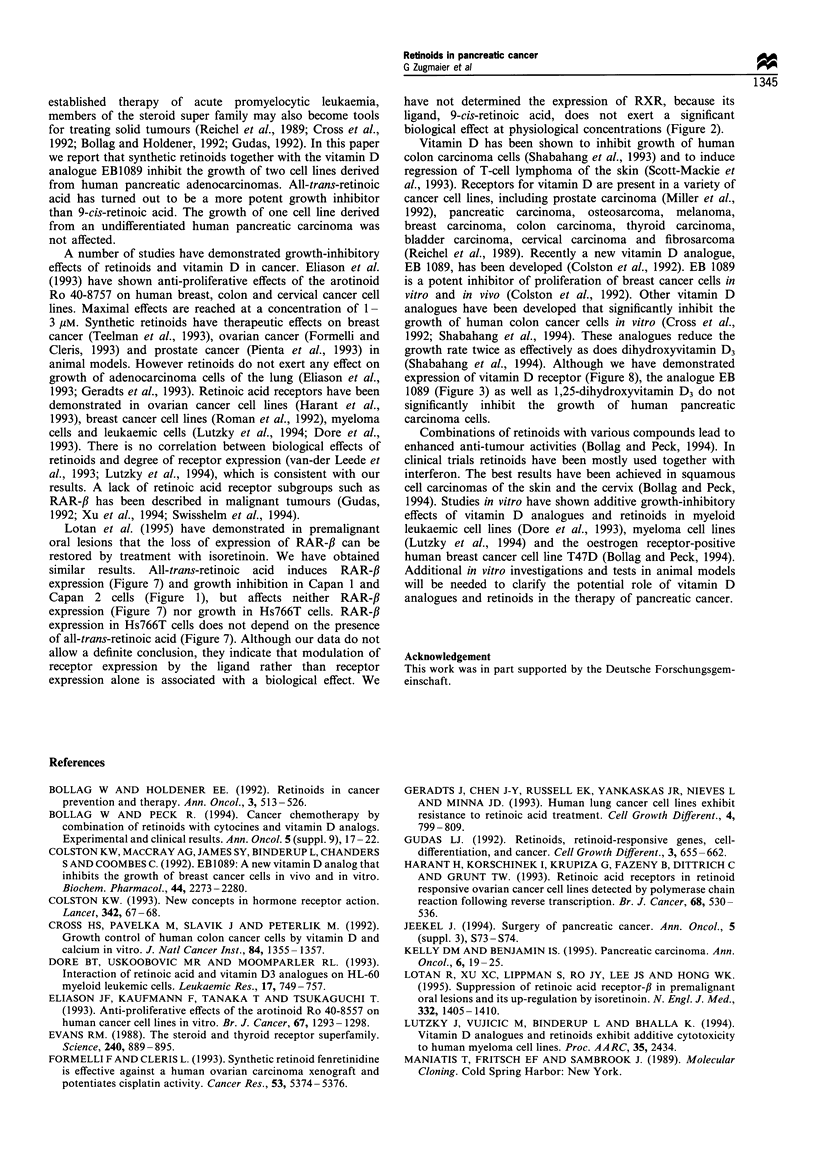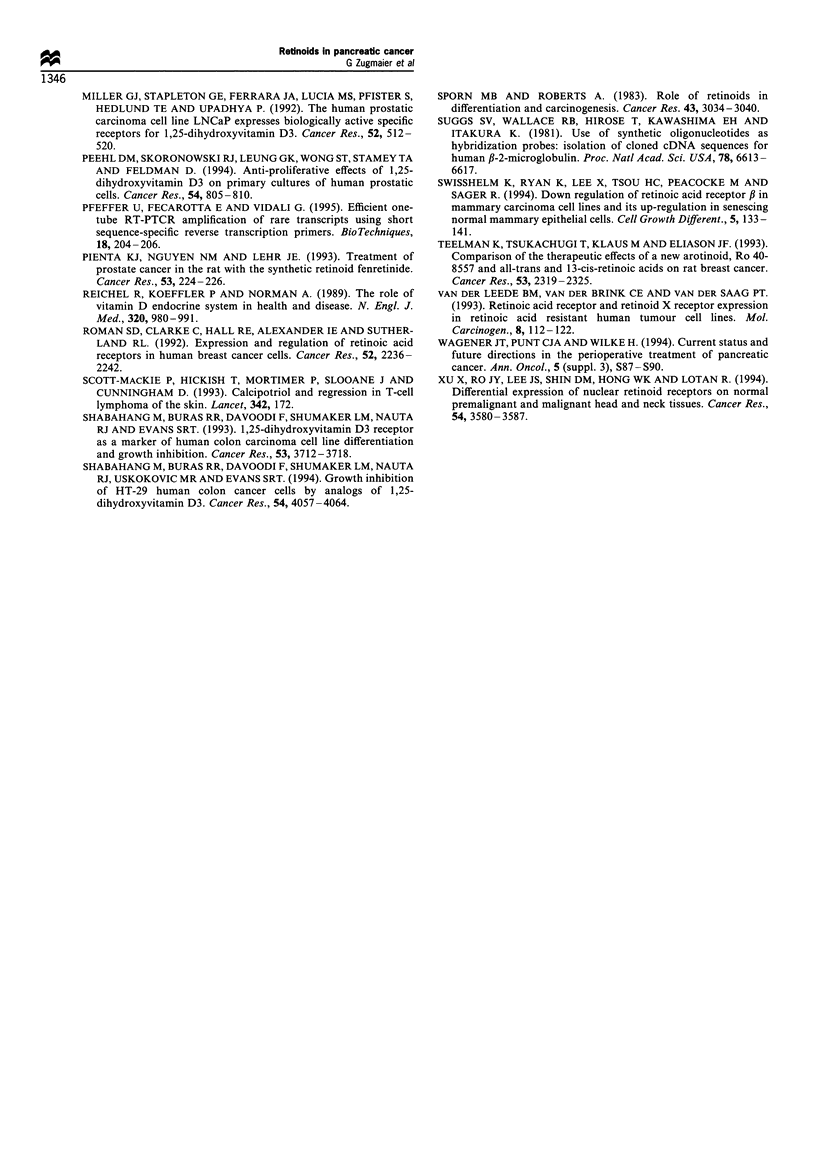# Growth-inhibitory effects of vitamin D analogues and retinoids on human pancreatic cancer cells.

**DOI:** 10.1038/bjc.1996.256

**Published:** 1996-06

**Authors:** G. Zugmaier, R. Jäger, B. Grage, M. M. Gottardis, K. Havemann, C. Knabbe

**Affiliations:** Department of Medical Oncology, Marburg University Medical Center, Germany.

## Abstract

**Images:**


British Journal of Cancer (1996) 73, 1341-1346
© 1996 Stockton Press All rights reserved 0007-0920/96 $12.00

### Growth-inhibitory effects of vitamin D analogues and retinoids on human pancreatic cancer cells

G Zugmaier[1], R Jäger[1], B Grage[2], MM Gottardis[3], K Havemann[1] and C Knabbe[2]

[1]*Department of Medical Oncology, Marburg University Medical Center, Baldinger Street, D-35033 Marburg, Germany;* [2]*Department of Clinical Chemistry, Medical Clinic, University Hospital, D-20246 Hamburg, Germany;* [3]*Department of Pharmacology, Ligand Inc., La Jolla, CA 92037, USA.*

**Summary** Retinoids and vitamin D are important factors that regulate cellular growth and differentiation. An additive growth-inhibitory effect of retinoids and vitamin D analogues has been demonstrated for human myeloma, leukaemic and breast cancer cells. We set out to study the effects of the vitamin D analogue EB1089 and the retinoids all-*trans*- and 9-*cis*-retinoic acid on the human pancreatic adenocarcinoma cell lines Capan 1 and Capan 2 and the undifferentiated pancreatic carcinoma cell line Hs766T. The cell lines investigated expressed vitamin D receptor, retinoic acid receptor (RAR)-$\alpha$ and $\gamma$ as determined by polymerase chain reaction after reverse transcription. RAR-$\beta$ was expressed only in Hs766T cells. Addition of all-*trans*-retinoic acid increased the amount of RAR-$\alpha$ mRNA in the three cell lines and induced RAR-$\beta$ mRNA in Capan 1 and Capan 2 cells. All-*trans*-retinoic acid at a concentration of 10 nM inhibited the growth of Capan 1 and Capan 2 cells by 40% relative to controls. 9-*cis*-Retinoic acid was less effective. Neither all-*trans*-retinoic acid nor 9-*cis*-retinoic acid affected the growth of Hs766T cells. EB1089, if added alone to the cells, did not significantly inhibit growth. However, the combination of 1 nM EB1089 with 10 nM all-*trans*-retinoic acid exerted a growth-inhibitory effect of 90% in Capan 1 cells and of 70% in Capan 2 cells. Our data suggest that vitamin D analogues together with retinoids inhibit the growth of human pancreatic cancer cells. However, *in vivo* studies are necessary to examine the potential use of retinoids and vitamin D analogues on pancreatic cancer.

**Keywords:** retinoids; reinoic acid; vitamin D; pancreatic cancer

Carcinoma of the pancreas is the fifth leading cause of death from malignant disease in Western society. In the United States incidence of pancreatic carcinoma has trebled in the last 50 years (Kelly and Benjamin, 1995). Pancreatic carcinoma is associated with an especially poor prognosis (Jeekel, 1994). Neither radiotherapy nor chemotherapy improve 5 year survival rates, which do not exceed 5% (Wagener *et al.*, 1994). Therefore new therapeutic modalities are essential for treating pancreatic carcinoma. There has been increasing evidence that the steroid hormones retinoic acid and vitamin D are naturally occurring agents controlling cellular differentiation and proliferation both in normal and malignant cells (Colston, 1993).

The biologically active form of vitamin D, 1,25-dihydroxy-vitamin $D_3$ [calcitriol-1,25(OH) $D_3$] exerts effects unrelated to calcium homeostasis such as inhibiting proliferation of cancer cells (Cross *et al.*, 1992). Receptors for vitamin D are present in a variety of cancer cells, including pancreatic adenocarcinoma (Reichel *et al.*, 1989). However, a major drawback to considering conventional vitamin D metabolites as therapeutic agents is the production of hypercalcaemia at doses more than a few micrograms per day (Reichel *et al.*, 1989). Recently a number of laboratories have developed synthetic vitamin D analogues that inhibit cancer cell growth, but have reduced calcaemic activity (Colston *et al.*, 1992; Shabahang *et al.*, 1994). One compound, EB 1089, which is characterised by a modified C17 side chain of the vitamin D molecule, inhibits the growth of breast cancer cells *in vitro* and *in vivo* (Colston *et al.*, 1992).

Retinoids are natural and synthetic derivatives to vitamin A (Bollag and Holdener, 1992). They elicit a large array of biological responses during morphogenesis and differentiation (Sporn and Roberts, 1983). Knowledge of the effects of these compounds has led to the assumption that retinoids may act as chemopreventive agents as well as inhibitors of tumour growth (Bollag and Holdener, 1992). Retinoids have been shown to depress tumour incidence and size in animal models (Gudas, 1992). Studies with cancer cells demonstrate growth inhibition induced by retinoids (Eliason *et al.*, 1993; Peehl *et al.*, 1994).

Combining retinoids with cytokines such as interferon leads to enhanced effects on tumours *in vitro* and *in vivo* (Bollag and Peck, 1994). Additionally, recent investigations have shown that retinoids together with vitamin D analogues induce additive growth inhibition of myeloma cells, leukaemic cells and breast cancer cells (Dore *et al.*, 1993; Lutzky *et al.*, 1994; Bollag and Peck, 1994).

We set out to study the effects of the retinoids all-*trans*-retinoic acid, 9-*cis*-retinoic acid and the vitamin D analogue EB1089 on three human pancreatic cell lines, two were derived from adenocarcinomas and one was derived from an undifferentiated carcinoma.

### Materials and methods

### *Cell culture*

NRK fibroblasts and the human pancreatic cancer cell lines Capan 1, Capan 2 and Hs766T were received from the American Tissue Type Culture Collection (ATCC, Rockville, MD, USA). Cell lines were cultivated in RPMI medium (Gibco, UK) supplemented with 10% heat-inactivated fetal bovine serum (FBS; Gibco, UK) and routinely tested for mycoplasma contamination.

### *Retinoids and vitamin D*

All-*trans*-retinoic acid, 9-*cis*-retinoic acid were a generous gift from Hoffman La Roche (Basle, Switzerland). The vitamin D analogue EB1089 was a generous gift from Leo pharmaceutical (Copenhagen, Denmark).

### *Proliferation assays*

Cells were plated into 24-well-plates (Costar) in RPMI medium containing 10% FBS. Approximately 2000-5000 cells per well were added in 1 ml of medium. After 24 h

Correspondence: C Knabbe
Received 29 June 1995; revised 22 December 1995; accepted 5 January 1996

retinoic acid or vitamin D analogues were added at various concentrations. Three to six wells were run at each data point. Cultures were allowed to grow in 5% carbon dioxide at 37°C. L-Glutamine was added every 2 days. After 2, 4, 7 and 9 days cells were detached with trypsin EDTA (Gibco, BRL) and their number was estimated using a Coulter cell counter (Coulter). Viability had been determined by trypan blue exclusion before cells were counted.

*RNA isolation*

Total cellular RNA was isolated by the phenol hot procedure (Maniatis *et al.*, 1989).

*Vitamin D receptor (VDR)*

Reverse transcription: 1 μg (7.5 μl) of total RNA was denatured at 70°C for 10 min. Then RNA was transcribed in RT-buffer (10 mM Tris pH 8.3, 50 mM potassium chloride, 1.5 mM magnesium chloride), 625 μM each dNTP, one unit of RNasin (Boehringer), 10 mM dithiothreitol, 1 μg random primer and five units of reverse transcriptase with a final total volume of 30 μl. The reaction mixture was incubated for 1 h at 37°C and for 10 min at 90°C. Polymerase chain reaction: 1.5 μl of cDNA product was amplified in 30 μl of 10 mM Tris pH 8.3, 50 mM potassium chloride, 1.5 mM magnesium chloride, 200 μM each dNTP, 0.5 μM each primer and 1.25 U *Taq* polymerase (Boehringer). Thirty cycles were performed, each consisting of 5 min at 95°C, 1 min at 58°C and 2 min at 73°C. PCR products were loaded on an agarose gel and stained with 1.5% ethidium bromide. The following primers were used (Evans, 1988):

Sense VDR: 5′-CTCCAGTTCGTGTGAATGATGG
Antisense: 5′-TTGTAGTCTTGGTTGCCACAGG

*RAR-α*

Reverse transcription and PCR were performed as described by Pfeffer *et al.* (1995).

*Reverse transcription* A 100 ng aliquot of total RNA, 25 pmol of a short sequence-specific transcription primer (5′-GGTTCAGGGTCAG, bp 1094–1082), 25 units of AMV reverse transcriptase (Boehringer, Mannheim), 1 mM each dNTP in a total volume of 20 μl in PCR buffer (10 mM Tris-HCL pH 8.3, 1.5 mM magnesium chloride, 50 mM potassium chloride, 0.005% Tween 20, 0.005% NP40) were incubated for 10 min at 25°C and for 45 min at 42°C. PCR was performed in the same buffer on a mini cycler (MJ Research) in 35 cycles consisting of 30 s at 94, 65, and 72°C each. The following primers were used:

Sense: 5′-ACCCCCTCTTACCCCGCATCTACAAG (bp 460 –484)
Antisense: 5′-CCGTCTGAGAAAGTCATGGTGTC (bp 1092 –1070)

Products were separated by standard agarose gel electrophoresis and stained with ethidium bromide.

*RAR-β and RAR-γ*

*Reverse transcription* A 200 ng aliquot of total RNA, five units reverse transcriptase with the following transcription primers:

RAR-β: 5′-GTCAAGGGTTCATGTCCTTC (bp 1593–1574)
RAR-γ: 5′-CGGCGCCGGGCGTACAGC (bp 1302–1285)

Incubation was performed at 52°C for 30 min, at 99°C for 1 min and at 4°C for 5 min.

Seminested PCR was performed essentially as described by Harant *et al.* (1993). In brief the first amplification was performed in a 20 μl reaction mix that was composed of 2 μl cDNA (equivalent to 500 ng of RNA), 2.5 μl of dNTP (Sigma, St Louis, MO, USA) (5 nmol of each dATP, dCTP, dGTP and dTTP), 2.5 μl each of 5′ and 3′ sequence primers (10 pmol $\mu l^{-1}$ each) and 5 μl of 10× buffer (100 mM Tris-HCl, pH 8.3, 500 mM potassium chloride, 15 mM magnesium chloride, 0.1% gelatin) and brought with water to a final volume of 20 μl. PCR was performed in a minicycler (MJ Research) for 35 cycles. The incubation times per cycle were 40 s at 94°C, 30 s at 60°C, and 1 min at 72°C with an extra 5 min for the last cycle. The following primers were used:

RAR-β sense: 5′-AGGAGACTTCGAAGCAAG (bp 822–839)
Antisense: 5′ - GTCAAGGGTTCATGTCCTTC (bp 1593 – 1574)
RAR-γ sense: 5′-GGAAGAAGGGTCACCTGA (bp 715–732)
Antisense: 5′-CGGCGCCGGGCGTACAGC (bp 1302–1285)

The second amplification consisted in 25 cycles as described above using the following degenerate primers:

RAR-β (bp 921–939), γ (bp 804–822)

A

TG C

Sense: 5′-CCTCGCTCTGCCAGCTGGG

Antisense primers are shown above

*$\beta_2$-Microglobulin*

Expression of the $\beta_2$-microglobulin mRNA was used as an internal control of the PCR reaction and reverse transcription: in contrast to the RAR-PCRs (see above) samples were taken after only 20 cycles, which is well before the time at which the PCR reaction reaches its plateau (data not shown). No signal without reverse transcription was obtained. The following primers were used:

Sense: 5′ - CAGCAAGGACTGGTCTTTCTATCTCTTGTA, corresponding to bases 201 – 230 of the cDNA (Suggs *et al.*, 1981)
Antisense: 5′ - GGAGCAACCTGCTCAGATACATCAAAA CATGG,
corresponding to bases 539–510 of the cDNA.

*Statistical analysis*

For each data point mean and standard deviation were calculated. Student's *t*-test was performed and a *P*-value of <0.01 was considered statistically different.

### Results

*Anchorage-dependent growth assays*

We studied the effects of all-*trans*-retinoic acid, 9-*cis*-retinoic acid and the vitamin D analogue EB1089 on the anchorage-independent growth of the human pancreatic adenocarcinoma cell lines Capan 1, Capan 2 and the undifferentiated human pancreatic carcinoma cell line Hs766T. Cells were incubated with concentrations of retinoids and EB1089 ranging from 1 pM to 1 μM. After 7 days of incubation cell number was quantified and compared with that in medium without added hormone. The anti-proliferative effects of all-*trans*-retinoic acid and 9-*cis*-retinoic acid were dose dependent (Figures 1 and 2). All-*trans*-retinioc acid at a concentration of 10 nM inhibited the growth of the pancreatic adenocarcinoma cell lines Capan 1 and Capan 2 by 40% relative to untreated controls (Figure 1). 9-*cis*-retinoic acid at the same concentration affected the growth by 25% as compared with untreated controls (Figure 2). The growth of the undifferentiated pancreatic carcinoma cell line Hs766T

was not affected by all-*trans*- or 9-*cis*-retinoic acid (data not shown). The vitamin D analogue EB 1089 had a maximal growth inhibitory effect of 25%, which was reached at a concentration of 1 nM in all three cell lines (Figure 3, shown for Capan 1 cells). Increasing concentrations of EB1089 did not enhance this effect (Figure 3), however, EB1089 potentiated the effects of all-*trans*- and 9-*cis*-retinoic acid (Figure 4). In the presence of 1 nM EB1089 all-*trans*-retinoic acid, at a concentration of 10 nM, induced an inhibition of 90% of growth in Capan 1 cells and an inhibition of 70% of growth in Capan 2 cells (Figure 4). 9-*cis*-Retinoic acid (10 nM) in the presence of 1 nM EB1089 was not as effective as all-*trans*-retinoic acid (Figure. 4). Vitamin $D_3$ had the same effects on growth as EB1089 (data not shown). All-*trans*-retinoic acid up to a concentration of 1000 nM did not reduce the growth of non-tumorigenic NRK cells to a statistically significant extent as compared with untreated controls (Figure 5).

*Expression of receptor mRNA*

Total RNA was extracted from the three human pancreatic cancer cell lines Capan 1, Capan 2 and Hs766T. RNA was transcribed into cDNA and then amplified using gene-specific primer pairs and polymerase chain reaction methodology. For RAR-α a specific amplification product could be easily detected corresponding in size to the product described by Pfeffer *et al.* (1995) in MCF-7 human breast cancer cells (Figure 6). No signal was obtained without reverse transcription, indicating that mRNA was specifically amplified. Because the first amplification revealed a very

**Figure 1** Effects of all-*trans*-retinoic acid on the anchorage-dependent growth of Capan 1 (■) and Capan 2 (□) cells. Cells were plated in triplicate in RPMI medium supplemented with 10% fetal bovine serum (FBS). After 24 h compounds were added. Fresh glutamine was added every 2 days. Cells were counted after 7–9 days. Standard deviations were 5–10%. Viability as determined by trypan blue exclusion was 90% in untreated and treated cells.

**Figure 3** Effect of the vitamin D analogue EB1089 on the anchorage-dependent growth of Capan 1 cells. Experiments were conducted as described in Figure 1.

**Figure 2** Effects of 9-*cis*-retinoic acid on the anchorage-dependent growth of Capan 1 (■) and Capan 2 (□) cells. Experiments were conducted as described in Figure 1.

**Figure 4** Inhibitory effects of all-*trans*-retinoic acid and 9-*cis* retinoic acid on the anchorage-dependent growth of Capan 1 (■) and Capan 2 (□) cells in the presence of 1 nM EB1089. Experiments were conducted as described in Figure 1.

weak signal of RAR-$\beta$ and RAR-$\gamma$ (data not shown), we applied a seminested PCR as second amplification step for detection of the mRNA of these two receptors. Using this approach the three cell lines showed a strong signal of RAR-$\gamma$ mRNA (Figure 6) corresponding to the specific product described earlier in ovarian cancer cells (Harant *et al*., 1993). In contrast, we found differential expression of RAR-$\beta$ mRNA, which could be detected only in Hs766T cells, matching the product described previously (Harant *et al*., 1993) but neither in Capan 1 nor in Capan 2 cells (Figure 6). Addition of 10 nM all-*trans*-retinoic acid induced RAR-$\beta$ expression in Capan 1 (Figure 7) and Capan 2 cells (not shown) but did not affect RAR-$\beta$ expression in Hs766T cells (Figure 7). Expression of $\beta$2-microglobulin mRNA measured after only 20 cycles of amplification was used as an internal control that showed a similar intensity in all samples tested, suggesting quantitative comparable PCR reactions (Figure 7). For expression of vitamin D receptor mRNA a signal was obtained in all cell lines using PCR after reverse transcription (Figure 8), which was the size expected from the cDNA sequence published earlier (Evans, 1988).

**Figure 5** Effects of all-*trans*-retinoic acid on non-tumorigenic NRK cells. Experiments were conducted as described in Figure 1.

**Figure 6** Detection of retinoic acid receptor mRNA by PCR after reverse transcription (+RT) and without reverse transcription (−RT). For detection of RAR-$\beta$ and $\gamma$ a seminested method was applied.

### Discussion

Pancreatic cancer is poorly influenced by chemotherapy (Jeekel, 1994), thus new therapeutic modalities are required to improve long-term survival of patients (Wagener *et al*., 1994). Since administration of retinoids has become an

**Figure 7** Effect of all-*trans*-retinoic acid on the expression of RAR-$\alpha$ and $\beta$ mRNA detected by PCR after reverse transcription. Cells had been incubated with 10 nM all-*trans*-retinoic acid for 2 days (+RA) as compared with untreated controls (−RA). For comparison the expression of the $\beta_2$-microglobulin mRNA was determined in the same samples.

**Figure 8** Detection of vitamin D receptor (VDR) mRNA by PCR after reverse transcription.

established therapy of acute promyelocytic leukaemia, members of the steroid super family may also become tools for treating solid tumours (Reichel *et al.*, 1989; Cross *et al.*, 1992; Bollag and Holdener, 1992; Gudas, 1992). In this paper we report that synthetic retinoids together with the vitamin D analogue EB1089 inhibit the growth of two cell lines derived from human pancreatic adenocarcinomas. All-*trans*-retinoic acid has turned out to be a more potent growth inhibitor than 9-*cis*-retinoic acid. The growth of one cell line derived from an undifferentiated human pancreatic carcinoma was not affected.

A number of studies have demonstrated growth-inhibitory effects of retinoids and vitamin D in cancer. Eliason *et al.* (1993) have shown anti-proliferative effects of the arotinoid Ro 40-8757 on human breast, colon and cervical cancer cell lines. Maximal effects are reached at a concentration of 1–3 $\mu$M. Synthetic retinoids have therapeutic effects on breast cancer (Teelman *et al.*, 1993), ovarian cancer (Formelli and Cleris, 1993) and prostate cancer (Pienta *et al.*, 1993) in animal models. However retinoids do not exert any effect on growth of adenocarcinoma cells of the lung (Eliason *et al.*, 1993; Geradts *et al.*, 1993). Retinoic acid receptors have been demonstrated in ovarian cancer cell lines (Harant *et al.*, 1993), breast cancer cell lines (Roman *et al.*, 1992), myeloma cells and leukaemic cells (Lutzky *et al.*, 1994; Dore *et al.*, 1993). There is no correlation between biological effects of retinoids and degree of receptor expression (van-der Leede *et al.*, 1993; Lutzky *et al.*, 1994), which is consistent with our results. A lack of retinoic acid receptor subgroups such as RAR-$\beta$ has been described in malignant tumours (Gudas, 1992; Xu *et al.*, 1994; Swisshelm *et al.*, 1994).

Lotan *et al.* (1995) have demonstrated in premalignant oral lesions that the loss of expression of RAR-$\beta$ can be restored by treatment with isoretinoin. We have obtained similar results. All-*trans*-retinoic acid induces RAR-$\beta$ expression (Figure 7) and growth inhibition in Capan 1 and Capan 2 cells (Figure 1), but affects neither RAR-$\beta$ expression (Figure 7) nor growth in Hs766T cells. RAR-$\beta$ expression in Hs766T cells does not depend on the presence of all-*trans*-retinoic acid (Figure 7). Although our data do not allow a definite conclusion, they indicate that modulation of receptor expression by the ligand rather than receptor expression alone is associated with a biological effect. We have not determined the expression of RXR, because its ligand, 9-*cis*-retinoic acid, does not exert a significant biological effect at physiological concentrations (Figure 2).

Vitamin D has been shown to inhibit growth of human colon carcinoma cells (Shabahang *et al.*, 1993) and to induce regression of T-cell lymphoma of the skin (Scott-Mackie *et al.*, 1993). Receptors for vitamin D are present in a variety of cancer cell lines, including prostate carcinoma (Miller *et al.*, 1992), pancreatic carcinoma, osteosarcoma, melanoma, breast carcinoma, colon carcinoma, thyroid carcinoma, bladder carcinoma, cervical carcinoma and fibrosarcoma (Reichel *et al.*, 1989). Recently a new vitamin D analogue, EB 1089, has been developed (Colston *et al.*, 1992). EB 1089 is a potent inhibitor of proliferation of breast cancer cells *in vitro* and *in vivo* (Colston *et al.*, 1992). Other vitamin D analogues have been developed that significantly inhibit the growth of human colon cancer cells *in vitro* (Cross *et al.*, 1992; Shabahang *et al.*, 1994). These analogues reduce the growth rate twice as effectively as does dihydroxyvitamin $D_3$ (Shabahang *et al.*, 1994). Although we have demonstrated expression of vitamin D receptor (Figure 8), the analogue EB 1089 (Figure 3) as well as 1,25-dihydroxyvitamin $D_3$ do not significantly inhibit the growth of human pancreatic carcinoma cells.

Combinations of retinoids with various compounds lead to enhanced anti-tumour activities (Bollag and Peck, 1994). In clinical trials retinoids have been mostly used together with interferon. The best results have been achieved in squamous cell carcinomas of the skin and the cervix (Bollag and Peck, 1994). Studies *in vitro* have shown additive growth-inhibitory effects of vitamin D analogues and retinoids in myeloid leukaemic cell lines (Dore *et al.*, 1993), myeloma cell lines (Lutzky *et al.*, 1994) and the oestrogen receptor-positive human breast cancer cell line T47D (Bollag and Peck, 1994). Additional *in vitro* investigations and tests in animal models will be needed to clarify the potential role of vitamin D analogues and retinoids in the therapy of pancreatic cancer.

**Acknowledgement**
This work was in part supported by the Deutsche Forschungsgemeinschaft.

### References

BOLLAG W AND HOLDENER EE. (1992). Retinoids in cancer prevention and therapy. *Ann. Oncol.*, **3**, 513–526.

BOLLAG W AND PECK R. (1994). Cancer chemotherapy by combination of retinoids with cytocines and vitamin D analogs. Experimental and clinical results. *Ann. Oncol.* **5** (suppl. 9), 17–22.

COLSTON KW, MACCRAY AG, JAMES SY, BINDERUP L, CHANDERS S AND COOMBES C. (1992). EB1089: A new vitamin D analog that inhibits the growth of breast cancer cells in vivo and in vitro. *Biochem. Pharmacol.*, **44**, 2273–2280.

COLSTON KW. (1993). New concepts in hormone receptor action. *Lancet*, **342**, 67–68.

CROSS HS, PAVELKA M, SLAVIK J AND PETERLIK M. (1992). Growth control of human colon cancer cells by vitamin D and calcium in vitro. *J. Natl Cancer Inst.*, **84**, 1355–1357.

DORE BT, USKOOBOVIC MR AND MOOMPARLER RL. (1993). Interaction of retinoic acid and vitamin D3 analogues on HL-60 myeloid leukemic cells. *Leukaemic Res.*, **17**, 749–757.

ELIASON JF, KAUFMANN F, TANAKA T AND TSUKAGUCHI T. (1993). Anti-proliferative effects of the arotinoid Ro 40-8557 on human cancer cell lines in vitro. *Br. J. Cancer*, **67**, 1293–1298.

EVANS RM. (1988). The steroid and thyroid receptor superfamily. *Science*, **240**, 889–895.

FORMELLI F AND CLERIS L. (1993). Synthetic retinoid fenretinidine is effective against a human ovarian carcinoma xenograft and potentiates cisplatin activity. *Cancer Res.*, **53**, 5374–5376.

GERADTS J, CHEN J-Y, RUSSELL EK, YANKASKAS JR, NIEVES L AND MINNA JD. (1993). Human lung cancer cell lines exhibit resistance to retinoic acid treatment. *Cell Growth Different.*, **4**, 799–809.

GUDAS LJ. (1992). Retinoids, retinoid-responsive genes, cell-differentiation, and cancer. *Cell Growth Different.*, **3**, 655–662.

HARANT H, KORSCHINEK I, KRUPIZA G, FAZENY B, DITTRICH C AND GRUNT TW. (1993). Retinoic acid receptors in retinoid responsive ovarian cancer cell lines detected by polymerase chain reaction following reverse transcription. *Br. J. Cancer*, **68**, 530–536.

JEEKEL J. (1994). Surgery of pancreatic cancer. *Ann. Oncol.*, **5** (suppl. 3), S73–S74.

KELLY DM AND BENJAMIN IS. (1995). Pancreatic carcinoma. *Ann. Oncol.*, **6**, 19–25.

LOTAN R, XU XC, LIPPMAN S, RO JY, LEE JS AND HONG WK. (1995). Suppression of retinoic acid receptor-$\beta$ in premalignant oral lesions and its up-regulation by isoretinoin. *N. Engl. J. Med.*, **332**, 1405–1410.

LUTZKY J, VUJICIC M, BINDERUP L AND BHALLA K. (1994). Vitamin D analogues and retinoids exhibit additive cytotoxicity to human myeloma cell lines. *Proc. AARC*, **35**, 2434.

MANIATIS T, FRITSCH EF AND SAMBROOK J. (1989). *Molecular Cloning*. Cold Spring Harbor: New York.

MILLER GJ, STAPLETON GE, FERRARA JA, LUCIA MS, PFISTER S, HEDLUND TE AND UPADHYA P. (1992). The human prostatic carcinoma cell line LNCaP expresses biologically active specific receptors for 1,25-dihydroxyvitamin D3. *Cancer Res.*, **52**, 512–520.

PEEHL DM, SKORONOWSKI RJ, LEUNG GK, WONG ST, STAMEY TA AND FELDMAN D. (1994). Anti-proliferative effects of 1,25-dihydroxyvitamin D3 on primary cultures of human prostatic cells. *Cancer Res.*, **54**, 805–810.

PFEFFER U, FECAROTTA E AND VIDALI G. (1995). Efficient one-tube RT-PTCR amplification of rare transcripts using short sequence-specific reverse transcription primers. *BioTechniques*, **18**, 204–206.

PIENTA KJ, NGUYEN NM AND LEHR JE. (1993). Treatment of prostate cancer in the rat with the synthetic retinoid fenretinide. *Cancer Res.*, **53**, 224–226.

REICHEL R, KOEFFLER P AND NORMAN A. (1989). The role of vitamin D endocrine system in health and disease. *N. Engl. J. Med.*, **320**, 980–991.

ROMAN SD, CLARKE C, HALL RE, ALEXANDER IE AND SUTHERLAND RL. (1992). Expression and regulation of retinoic acid receptors in human breast cancer cells. *Cancer Res.*, **52**, 2236–2242.

SCOTT-MACKIE P, HICKISH T, MORTIMER P, SLOOANE J AND CUNNINGHAM D. (1993). Calcipotriol and regression in T-cell lymphoma of the skin. *Lancet*, **342**, 172.

SHABAHANG M, BURAS RR, DAVOODI F, SHUMAKER LM, NAUTA RJ AND EVANS SRT. (1993). 1,25-dihydroxyvitamin D3 receptor as a marker of human colon carcinoma cell line differentiation and growth inhibition. *Cancer Res.*, **53**, 3712–3718.

SHABAHANG M, BURAS RR, DAVOODI F, SHUMAKER LM, NAUTA RJ, USKOKOVIC MR AND EVANS SRT. (1994). Growth inhibition of HT-29 human colon cancer cells by analogs of 1,25-dihydroxyvitamin D3. *Cancer Res.*, **54**, 4057–4064.

SPORN MB AND ROBERTS A. (1983). Role of retinoids in differentiation and carcinogenesis. *Cancer Res.* **43**, 3034–3040.

SUGGS SV, WALLACE RB, HIROSE T, KAWASHIMA EH AND ITAKURA K. (1981). Use of synthetic oligonucleotides as hybridization probes: isolation of cloned cDNA sequences for human β-2-microglobulin. *Proc. Natl Acad. Sci. USA*, **78**, 6613–6617.

SWISSHELM K, RYAN K, LEE X, TSOU HC, PEACOCKE M AND SAGER R. (1994). Down regulation of retinoic acid receptor β in mammary carcinoma cell lines and its up-regulation in senescing normal mammary epithelial cells. *Cell Growth Different.*, **5**, 133–141.

TEELMAN K, TSUKACHUGI T, KLAUS M AND ELIASON JF. (1993). Comparison of the therapeutic effects of a new arotinoid, Ro 40-8557 and all-trans and 13-cis-retinoic acids on rat breast cancer. *Cancer Res.*, **53**, 2319–2325.

VAN DER LEEDE BM, VAN DER BRINK CE AND VAN DER SAAG PT. (1993). Retinoic acid receptor and retinoid X receptor expression in retinoic acid resistant human tumour cell lines. *Mol. Carcinogen.*, **8**, 112–122.

WAGENER JT, PUNT CJA AND WILKE H. (1994). Current status and future directions in the perioperative treatment of pancreatic cancer. *Ann. Oncol.*, **5** (suppl. 3), S87–S90.

XU X, RO JY, LEE JS, SHIN DM, HONG WK AND LOTAN R. (1994). Differential expression of nuclear retinoid receptors on normal premalignant and malignant head and neck tissues. *Cancer Res.*, **54**, 3580–3587.